# Accumulation of Pharmaceuticals, *Enterococcus*, and Resistance Genes in Soils Irrigated with Wastewater for Zero to 100 Years in Central Mexico

**DOI:** 10.1371/journal.pone.0045397

**Published:** 2012-09-25

**Authors:** Philipp Dalkmann, Melanie Broszat, Christina Siebe, Elisha Willaschek, Tuerkan Sakinc, Johannes Huebner, Wulf Amelung, Elisabeth Grohmann, Jan Siemens

**Affiliations:** 1 Institute of Crop Science and Resource Conservation – Soil Science and Soil Ecology, University of Bonn, Bonn, Germany; 2 Department of Infectious Diseases, University Hospital Freiburg, Freiburg, Germany; 3 Faculty for Biology, Microbiology, Albert-Ludwigs-University Freiburg, Freiburg, Germany; 4 Instituto de Geología, Universidad Nacional Autónoma de México, México D.F., México; Auburn University, United States of America

## Abstract

Irrigation with wastewater releases pharmaceuticals, pathogenic bacteria, and resistance genes, but little is known about the accumulation of these contaminants in the environment when wastewater is applied for decades. We sampled a chronosequence of soils that were variously irrigated with wastewater from zero up to 100 years in the Mezquital Valley, Mexico, and investigated the accumulation of ciprofloxacin, enrofloxacin, sulfamethoxazole, trimethoprim, clarithromycin, carbamazepine, bezafibrate, naproxen, diclofenac, as well as the occurrence of *Enterococcus* spp., and *sul* and *qnr* resistance genes. Total concentrations of ciprofloxacin, sulfamethoxazole, and carbamazepine increased with irrigation duration reaching 95% of their upper limit of 1.4 µg/kg (ciprofloxacin), 4.3 µg/kg (sulfamethoxazole), and 5.4 µg/kg (carbamazepine) in soils irrigated for 19–28 years. Accumulation was soil-type-specific, with largest accumulation rates in Leptosols and no time-trend in Vertisols. Acidic pharmaceuticals (diclofenac, naproxen, bezafibrate) were not retained and thus did not accumulate in soils. We did not detect *qnrA* genes, but *qnrS* and *qnrB* genes were found in two of the irrigated soils. Relative concentrations of *sul1* genes in irrigated soils were two orders of magnitude larger (3.15×10^−3^±0.22×10^−3^ copies/16S rDNA) than in non-irrigated soils (4.35×10^−5^±1.00×10^−5^ copies/16S rDNA), while those of *sul2* exceeded the ones in non-irrigated soils still by a factor of 22 (6.61×10^–4^±0.59×10^−4^ versus 2.99×10^−5^±0.26×10^−5^ copies/16S rDNA). Absolute numbers of *sul* genes continued to increase with prolonging irrigation together with *Enterococcus* spp. 23S rDNA and total 16S rDNA contents. Increasing total concentrations of antibiotics in soil are not accompanied by increasing relative abundances of resistance genes. Nevertheless, wastewater irrigation enlarges the absolute concentration of resistance genes in soils due to a long-term increase in total microbial biomass.

## Introduction

The coexistence of antibiotics, pathogenic bacteria, and resistance determinants in the environment raises concerns that antibiotic resistance genes are mobilized from and disseminated into the environmental resistome and transferred to bacteria that are potentially pathogenic to humans [1], [2], [3]. These risks might be particularly high for agricultural fields that are irrigated with wastewater, which receive regular inputs of antibiotics, bacteria, and resistance genes similar to manured soils (e.g., [4]). Nevertheless, climate change, population growth, as well as urbanization increase the pressure on water resources in many regions of the world [5], [6]. Thus, the pressure of re-using wastewater for irrigation and food production (e.g., [7], [8]) or to recharge groundwater for drinking water supply (e.g., [9], [10]) is increasing rather than decreasing, with unforeseen risks in the long-term.

One of the largest wastewater irrigation areas worldwide can be found in the Mezquital Valley receiving wastewater from the Mexico City Metropolitan Area (MCMA) [11]. Gibson et al. [12], [13] as well as Siemens et al. [14] already documented that this wastewater contains a wide range of pharmaceuticals. Consequently, pharmaceuticals have also been found in soils irrigated with MCMA wastewater [13], [15]. Reports on the occurrence of pharmaceuticals in wastewater-irrigated soils in Colorado, USA [16], Braunschweig, Germany [17], Hebei, China [18], and the metropolitan area of Paris, France [19] illustrate that the contamination of soils with wastewater-derived pharmaceuticals is not limited to Mexico, but a global phenomenon.

Along with pharmaceuticals, certain bacterial species, part of which are pathogenic or resistant to antibiotic agents or both, are released into wastewater irrigation channels and fields (e.g., [20], [21], [22]). Bacteria resistant to antibiotics have been isolated previously from wastewater-irrigated fields in India [23]. *Enterococcus spp.* are commonly used as hygienic indicator in the environment since they mainly originate from animal and human faeces [24]. In wastewater the predominant species are *E. faecalis*, *E. faecium* and *E. hirae* [25], [26], [27], [28]. *E. faecalis* and *E. faecium* are currently also the third most commonly isolated nosocomial pathogens worldwide and the second most common nosocomial pathogens isolated from intensive care patients worldwide [29], [30], [31], [32]. Acquired antibiotic resistances in enterococci are reported in an increasing number of hospital isolates [29]. Whether the abundance of such organisms increases in soil with repeated wastewater application has not been studied yet, to our knowledge.

Several investigations have studied the occurrence of antibiotic resistance genes in different environmental compartments including wastewater, wastewater lagoons, surface waters, river sediments, pristine soils, and manured soils (e.g., [33], [34], [35], [36], [37], [38], [39]). Very limited information is, however, available on the presence of antibiotic resistance genes in soils to which wastewater or biosolids have been applied [40]. All these studies reached the conclusion that antibiotic resistance genes are comparable to “emerging contaminants” that need to be further studied. It is important to note, however, that resistance genes do not only enter the environment with livestock or human waste, but are common also in rather pristine environments, especially in soils (e.g., [1], [41]). Recent laboratory experiments with *E. coli* and *Salmonella enterica* showed that a selection of antibiotic resistance occurs at very small sub-inhibitory concentrations of antibiotics that are usually encountered in polluted environments [42]. Of particular concern for public health is the selection of genes conferring resistance towards sulfonamides and fluoroquinolones. *Sul* resistance genes (*sul1*, *sul2*) conferring resistance to sulfonamides occur in a wide range of bacterial species, because they are often encoded on transposable elements of conjugative or mobilizable broad-host-range plasmids [38], [43], [44]. The fluoroquinolone resistance genes *qnrA*, *qnrB*, and *qnrS* are also often plasmid-encoded, threatening to accelerate the spread of resistance through horizontal transfer [37]. Quinolone resistance is on the rise, especially among the *Enterobacteriacae* [45], [46]. The relevance of these resistance genes for public health is reflected by the fact that *sul1* and *sul2* have been detected in a variety of clinical *Enterobacteriaceae* isolates (e.g., [47]), the *qnr* genes in many clinical *Klebsiella pneumoniae* isolates and in *E. faecalis* [48], [49], [50], [51].

In summary, the presence of pharmaceuticals in wastewater-irrigated soils has been documented for several sites. However, it is currently unclear whether and to which degree continuous irrigation with wastewater may lead to a long-term accumulation of these agents in soil. We hypothesize that similar to the changes of natural organic matter with prolonged land-use (e.g., [52], [53], [54]) a steady state in soil is approached between input with wastewater, decay, and output of pharmaceuticals with drainage and harvested crops (assuming constant input). Yet, our knowledge on how fast and to which degree this steady state is reached after prolonged wastewater irrigation is unknown. As the accumulation of pharmaceuticals may be associated with the accumulation of resistance genes derived from wastewater or the “native” soil resistome, we included the analysis of resistance genes and *Enterococcus* ssp. into our assessment of soil contamination in the Mezquital Valley, Mexico. The studied soils received untreated MCMA wastewater from zero to 100 years; soils under rainfed irrigation served as control.

## Materials and Methods

### Soils

Over the past century the irrigated area in the Mezquital Valley increased due to the expansion of the MCMA. We chose sites with different duration of irrigation with untreated wastewater (0, 1.5, 3, 6, 8, 11, 12, 13.5, 23, 35, 50, 85, and 100 years) for our study, each of which was either sampled between June and August 2009 or in March 2011. The second sampling campaign in March 2011 was necessary to increase the temporal resolution of the soil chronosequence particularly for the short irrigation periods (a detailed list of samples can be found in Table S1 in the supporting information, SI). Depending on their clay content and the thickness of the solum the soils in the Mezquital Valley have been classified as Leptosols, Vertisols, and Phaeozems [8] according to the World Reference Base for Soil Resources [55]. Soil properties are given in Table 1. All soils have been irrigated with MCMA wastewater, which has been well mixed especially over longer time periods because of the extensive pumping and diversion of wastewater within the MCMA and the Mezquital Valley irrigation system. Each individual field was subdivided into four parcels, two on the wastewater inflow side and two on the wastewater outflow side of the field. From each parcel a sample composed of twelve subsamples was taken with an auger at a depth of 0–30 cm. Soil samples were collected in plastic bags, transported to the laboratory in cooling bags (around 4°C) and stored at −21°C until extraction.

**Table 1:** Soil properties.

Soil type	Clay content^a^(range) [%]	Clay content^a^(mean) [%]	OC content^b^(range) [%]	OC content^b^(mean) [%]	pH (range)	pH (mean)
Leptosols (LP)	18.6–39.1	31.6	1.1–2.4	1.9	6.6–8.2	7.5
Phaeozems (PH)	15.6–31.9	22.6	1.2–2.7	1.8	6.8–8.0	7.3
Vertisols (VR)	28.9–54.4	43.5	1.6–2.6	2.2	6.3–7.7	7.1

a Data are from Siebe [73];

b organic carbon (OC) content.

### Pharmaceutical Agents

Based on consumption data of Mexico [56] and ecotoxicological relevance, research concentrated on the compounds ciprofloxacin, enrofloxacin, sulfamethoxazole, trimethoprim, clarithromycin, carbamazepine, bezafibrate, naproxen, and diclofenac, the standards of which were obtained from Sigma-Aldrich (Schnelldorf, Germany). Important physicochemical properties of these compounds are listed in Table 2. Isotope-labeled ciprofloxacin (carboxyl-^13^C_3_, quinoline-^15^N, ≥98% pure), enrofloxacin hydrochloride (ethyl-d_5_, ≥98% pure), sulfamethoxazole (ring-^13^C_6_, ≥98% pure), trimethoprim (methyl-^13^C_3_, ≥98% pure), and carbamazepine (phenyl-d_10_, ≥98% pure) were supplied by LGC Standards (Wesel, Germany) as internal standards. Labeled bezafibrate (phenyl-d_4_, >98% pure) and clarithromycin (methyl-d_3_, 98% pure) were obtained from Toronto Research Chemicals (North York, Canada). Labeled naproxen (methyl-d_3_, 98% pure) and diclofenac (phenyl-d_4_, 99% pure) were purchased from Dr. Ehrenstorfer (Augsburg, Germany).

**Table 2:** Compound properties and measurement details.

Compound	Watersolubilitya,b[g/L]	logP_ow_ b	K_OC_[L/kg]	pK_a_ b	Excretionrate^c^ [%]	PEC^d^[µg/L]	PrecursorIon [m/z]	DaughterIons [m/z]	CollisionEnergy [eV]
ciprofloxacin	0.5	1.63	3487^e^	6.4;8.7	20.0	0.30	332.09	245.06	24
								288.11	17
enrofloxacin	0.1	2.31	2179^e^	6.4;7.8	n.a.^g^	n.a.^g^	360.12	245.06	26
								316.16	18
sulfamethoxazole	2.8	0.66	219^f^	5.8;1.4	30.0	1.96	254.02	108.03	23
								155.97	15
trimethoprim	1.0	0.59	301^f^	7.0	80	1.05	291.10	123.06	32
								230.09	23
clarithromycin	460.0	2.81	64.4^b^	13.1;8.2	25.0	0.09	748.43	158.00	28
								590.23	18
carbamazepine	0.2	1.90	363^f^	13.9	3.0	0.03	237.08	179.08	34
								194.10	20
naproxen	15.0	2.88	302^f^	4.8	5.5	0.43	231.08	170.08	26
								185.10	12
diclofenac	2.3	4.55	245^f^	4.2	5.5	0.06	296.00	214.02	35
								250.02	13
bezafibrate	140.0	2.50	398^f^	3.3	50.0	0.10	362.08	121.06	29
								138.97	26

a at pH 7 and 25°C;

b Data are from SciFinder Database (https://scifinder.cas.org), accessed May 2, 2012;

c Data are from Verlicchi et al. [74];

d Predicted Environmental Concentration (including excretion rate, mean 2003/2004);

e Data are from Figuero-Diva et al. [75];

f Data are from Barron et al. [76];

g not available.

### Extraction of Pharmaceuticals from Soil and their Detection

Soil samples were lyophilized and sieved to a grain size <2 mm. We distributed ten grams of dry matter (DM) of each soil into borosilicate centrifuge glasses. The extraction of an easily extractable, “bioaccessible” compound fraction was performed with 25 mL of a 0.01 M CaCl_2_ solution [57]. To assess the strongly bound, sequestered fraction of pharmaceuticals in soil, the CaCl_2_-extracted soil samples were lyophilized again and extracted via accelerated solvent extraction (ASE). We combined two different solvents for the extraction to account for the different physico-chemical properties of the pharmaceuticals (Table 2). We used an aqueous 50 mM phosphoric acid:acetonitrile solution (50∶50, v/v; according to Golet et al. [58] and a methanol:water solution (50∶50, v/v; according to Gobel et al. [59]). Extraction recoveries of the extraction method varied between 54–95% (Table S2 in the SI).

The analysis of pharmaceutical concentrations in soil extracts was performed with liquid chromatography tandem mass spectrometry (LC-MS/MS). Routine limit of quantification (RLOQ = lowest concentration of standard used) were 42 ng/kg dry soil in the CaCl_2_-extracts (naproxen: 428 ng/kg) and 57 ng/kg in the ASE-extracts (naproxen: 570 ng/kg). A detailed description of the extraction procedure and the analyses of pharmaceutical concentrations can be found in Text S1, Text S2, and Table S3 in the SI.

### Quantification of Antibiotic Resistance Genes and Enterococci in Soil Samples

Aliquots of soil samples from fields of the chronosequence irrigated for 0, 1.5, 3, 6, 8, 85, and 100 years were analyzed by real-time qPCR, in the following denominated as qPCR. We did not analyze soil samples of other irrigation durations specified above, because these samples were transported with 24 h delay from Mexico to Germany by the airline and were partly thawed by arrival. For each composite sample we pooled 10 g of the four parcels. Total DNA was extracted from 500 mg soil using the NucleoSpin® Soil kit according to the manufacturer’s protocol (Macherey-Nagel, Düren, Germany). Absolute quantifications of 16S rDNA, *sul1*, *sul2*, *qnrA*, *qnrB*, *qnrS*, and *Enterococcus* spp. 23S rRNA genes were performed with serial diluted exogenous standards that consisted of purified PCR products. PCR products were purified with QIAquick Gel Extraction Kit (Qiagen, Hilden, Germany).

Quantification of absolute target gene numbers was carried out using the Light-Cycler 480 (Roche Diagnostics, Mannheim, Germany). The limit of quantification (LOQ) for *sul1*, *sul2*, *qnrB*, and *qnrS* was 10 gene copies/reaction. For 16S rDNA, *Enterococcus* spp. and *qnrA* the limit of quantification equaled 100 gene copies/reaction. Reagents and programs for qPCR are listed in Table S4 and S5 of the SI. A detailed description of the method can be found in Text S3 in the SI.

### Data Evaluation

The substance amounts in the CaCl_2_- and ASE-extracts were summed to determine the total extractable pharmaceuticals in the soils. A model of exponential dissipation at constant inflow (equation 1) was fitted to the measured total soil concentrations,
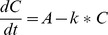
(1)with *C* denoting the concentration in soil [µg/kg], *t* denoting the irrigation period [yr], *A* the compound inflow [µg/kg*yr], and *k* denoting the dissipation rate constant [1/yr]. In this context “dissipation” includes all processes leading to a decrease of extractable concentrations in soil, such as biodegradation, photolysis, volatilization, leaching, plant uptake, and sequestration in non-extractable residues. Following integration, the concentration of pharmaceuticals in soil at a given point of time equals (eq. 2)
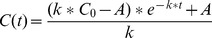
(2)with C(t) denoting the concentration [µg/kg] at a given point of time and C0 the concentration at the start of irrigation [µg/kg].

Predicted environmental concentrations (PEC, µg/L) of the pharmaceuticals in wastewater were calculated according to equation 3 (after [14]),
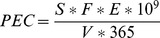
(3)with *S* denoting the active moiety of the pharmaceutical sold in Mexico [kg/yr], *V* denoting the water flow through the MCMA sewer system [L/d], *F* as the fraction of the Mexican population living in the MCMA (0.19), and *E* as the excretion rate of the respective pharmaceutical (Table 2).

Correlations between irrigation time of sites and their concentrations of resistance genes, *Enterococcus* 23S rDNA genes, and total concentrations of 16S rDNA genes were analyzed for statistical significance with the Spearman rank correlation coefficient and Kendalls tau using the Statistica 8.0 software (Statsoft, Tulsa, USA).

## Results and Discussion

### Pharmaceuticals

Soils under rain-fed agriculture contained on average 0.2 µg/kg of the analyzed pharmaceuticals. Potential reasons for the detection of these chemicals in non-irrigated soils are for example the deposition of wastewater aerosol close to irrigation channels, deposition of soil material derived from neighboring irrigated sites by wind erosion, or the transport of soil material between fields with farm machinery. Differences between the concentration levels of pharmaceuticals, their disposition to accumulation, and their bioaccessibility were observed between non-irrigated soils and irrigated soils as well as among irrigated soils. Total extractable concentrations of pharmaceuticals in irrigated soils averaged over the four parcels of each plot reached a maximum of 8.38 µg/kg soil (Table S6 in the SI). Concentrations of the acidic pharmaceuticals naproxen, diclofenac, and bezafibrate ranged between 0.51–3.06 µg/kg, 0.10–0.54 µg/kg, and <LOD−1.07 µg/kg, respectively. Similar results were obtained by Gibson et al. [13] for naproxen (0.27–0.61 µg/kg) and diclofenac (<LOD) for soils of the Mezquital Valley. The concentrations of these acidic pharmaceuticals in soils did not increase with increasing time of wastewater irrigation (Table S6). This lack of accumulation confirms the results of Siemens et al. [14], [60] and Durán-Álvarez et al. [61] regarding the poor retardation of these compounds in soil. Due to the alkaline pH values of the wastewater and the receiving soils that exceed the acid dissociation constants of naproxen, diclofenac, and bezafibrate, these compounds occur as negatively charged species. This negative charge counteracts sorption to negatively charged clays and moieties of soil organic matter [14], also expressed by small K_OC_ values at near neutral pH (Table 2). In the column experiments of Siemens et al. [60] with soil from the Mezquital Valley degradation of naproxen could not be detected while bezafibrate transport could be described with a first order degradation rate constant of 0.033±0.03/h.

The antibiotic sulfamethoxazole and the anticonvulsant carbamazepine were detected with the largest range of concentrations in irrigated soils, spanning from 0.98–5.96 µg/kg for sulfamethoxazole and from 1.49–8.38 µg/kg for carbamazepine. Carbamazepine concentrations are in accordance with the findings of Gibson et al. [13], who detected concentrations in the A horizon of soils in the Mezquital Valley ranging from 2.6 to 7.5 µg/kg. Total soil concentrations of the antibiotics trimethoprim (0.13–2.44 µg/kg) and ciprofloxacin (0.35–2.62 µg/kg) were smaller, despite the fact that their predicted concentrations in wastewater (PEC: 1.05 and 0.30 µg/L, respectively) were larger than for carbamazepine (PEC: 0.03 µg/L). For clarithromycin, the largest concentration found in our study was 5.43 µg/kg, but this concentration was only present in one plot. For the other plots, the concentrations of clarithromycin were generally smaller (<3 µg/kg), in line with comparably smaller predicted concentrations of this compound in wastewater (PEC: 0.09 µg/L). The veterinary antibiotic enrofloxacin was detected in small concentrations between <LOD and 1.21 µg/kg soil, suggesting that not only pharmaceuticals intended for human consumption reach the soils of the Mezquital Valley, but also veterinary pharmaceuticals.

**Figure 1:**
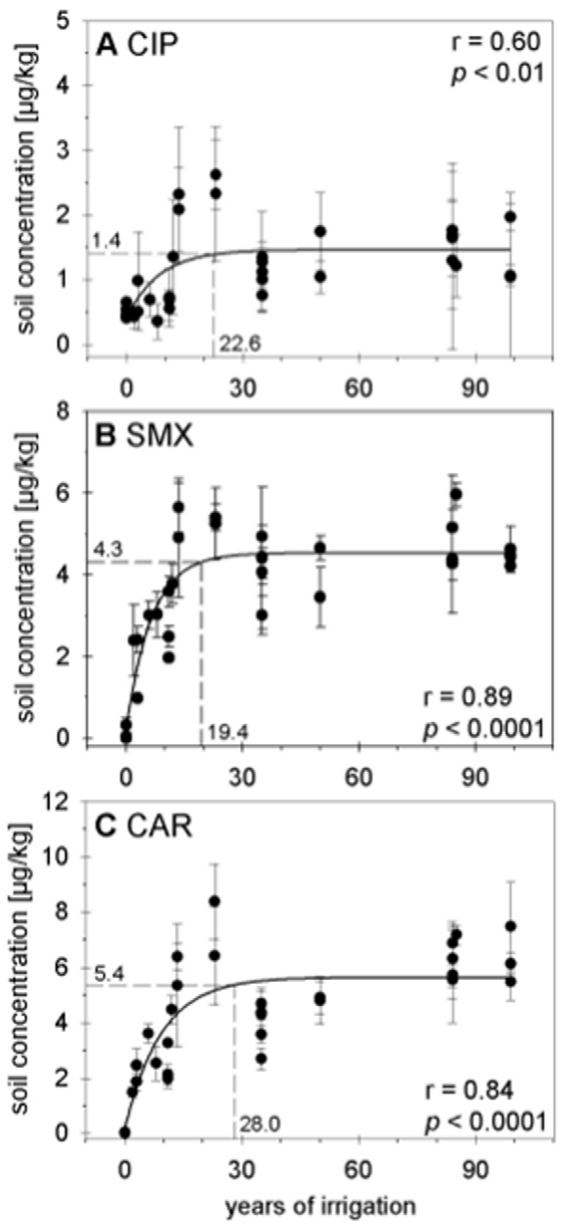
Concentrations of ciprofloxacin (CIP), sulfamethoxazole (SMX), and carbamazepine (CAR) in soils irrigated repeatedly with untreated wastewater. Irrigation took place for different numbers of years. The displayed total concentrations were calculated as the sum of CaCl_2_-extractable and ASE-extractable concentrations (Figure S1 in the supporting online information). Error bars indicate the standard deviation of concentrations in four quadrant parcels of individual fields. Dashed lines mark the irrigation time until 95% of the upper limit concentration is reached.

Total concentrations of sulfamethoxazole, ciprofloxacin, and carbamazepine in soils rapidly increased with increasing duration of wastewater irrigation until no further increase of concentration could be observed in soils irrigated for more than approximately 25 years (Figure 1A, 1B, 1C). The 22.6 years of time span for approaching nearly constant concentrations of total extractable ciprofloxacin in soil matches almost exactly the time since when this compound was on the market. It was introduced 1983 and approved by the U.S. Federal Drug Administration in 1987. The plateau of total extractable ciprofloxacin concentrations therefore might be influenced by the point of time when this drug was first released into the environment and not unequivocally indicate a steady state between input and dissipation. Given the long persistence of ciprofloxacin [62], the concentration of only 1.5 µg/kg that is approached after 25 years appears rather small. We suspect that this small concentration is explained by the strong sorption [63] and poor extractability of ciprofloxacin. Sorption of ciprofloxacin takes place predominantly via cation exchange or cation bridges (especially with Ca^2+^, [64]) or both. The large cation exchange capacity and Ca^2+^ saturation of the soils of the Mezquital Valley [65] therefore favor the sorption of ciprofloxacin. Moreover, ciprofloxacin occurs in its zwitterionic form at the neutral to slightly alkaline pH of the Mezquital Valley soils and this species is sorbed most effectively [63]. Among different soil types, Vertisols similar to those occurring in the Mezquital Valley sorbed ciprofloxacin most strongly in experiments of Vasudevan et al. [66].

In contrast to ciprofloxacin, sulfamethoxazole and carbamazepine were introduced into clinical practice much earlier (i.e., in the 1960’s). Nevertheless, we did not observe a prolonged accumulation of these two compounds for more than 19 and 28 years, respectively (Figure 1B, 1C). Moreover, the final concentrations of sulfamethoxazole (4.3 µg/kg) appear small given the large PEC of this compound in relation to PECs of other compounds (PEC: 1.96 µg/L). These rather small concentrations are probably not related to a strong binding or poor extractability, because also the log P_ow_ of sulfamethoxazole is low (0.66; Table 2). Instead, the short half live of only two days that Liu et al. [67] observed for sulfamethoxazole in non-sterile soil, but not in sterile soil suggests that small concentrations of this compound after long-term irrigation are a consequence of effective biodegradation. Overall, large input concentrations of sulfamethoxazole in combination with only moderate sorption and fast dissipation suggest that the plateau concentration of 4.3 µg/kg in the soils of the Mezquital Valley is likely reflecting a steady state equilibrium between input and dissipation.

Despite a more than 180 times smaller predicted concentration in wastewater of only 0.03 µg/L, carbamazepine reached a higher plateau concentration of 5.4 µg/kg in the Mezquital Valley soils over time (Figure 1C). This stronger accumulation in comparison to sulfamethoxazole was on the one hand favored by a smaller water solubility, which correlated with retention of pharmaceuticals in the Mezquital Valley soil in the transport study of Siemens et al. [60]. Large contents of soil organic matter in the topsoils of the Mezquital Valley favor the sorption of this neutral compound [68], which is also indicated by a correlation between organic carbon content and carbamazepine concentration reported by Gibson et al. [13] and a comparably high K_OC_ value (Table 2). On the other hand, and possibly most important for the observed accumulation of carbamazepine, is its pronounced recalcitrance against biodegradation [69], [70], [71].

**Figure 2:**
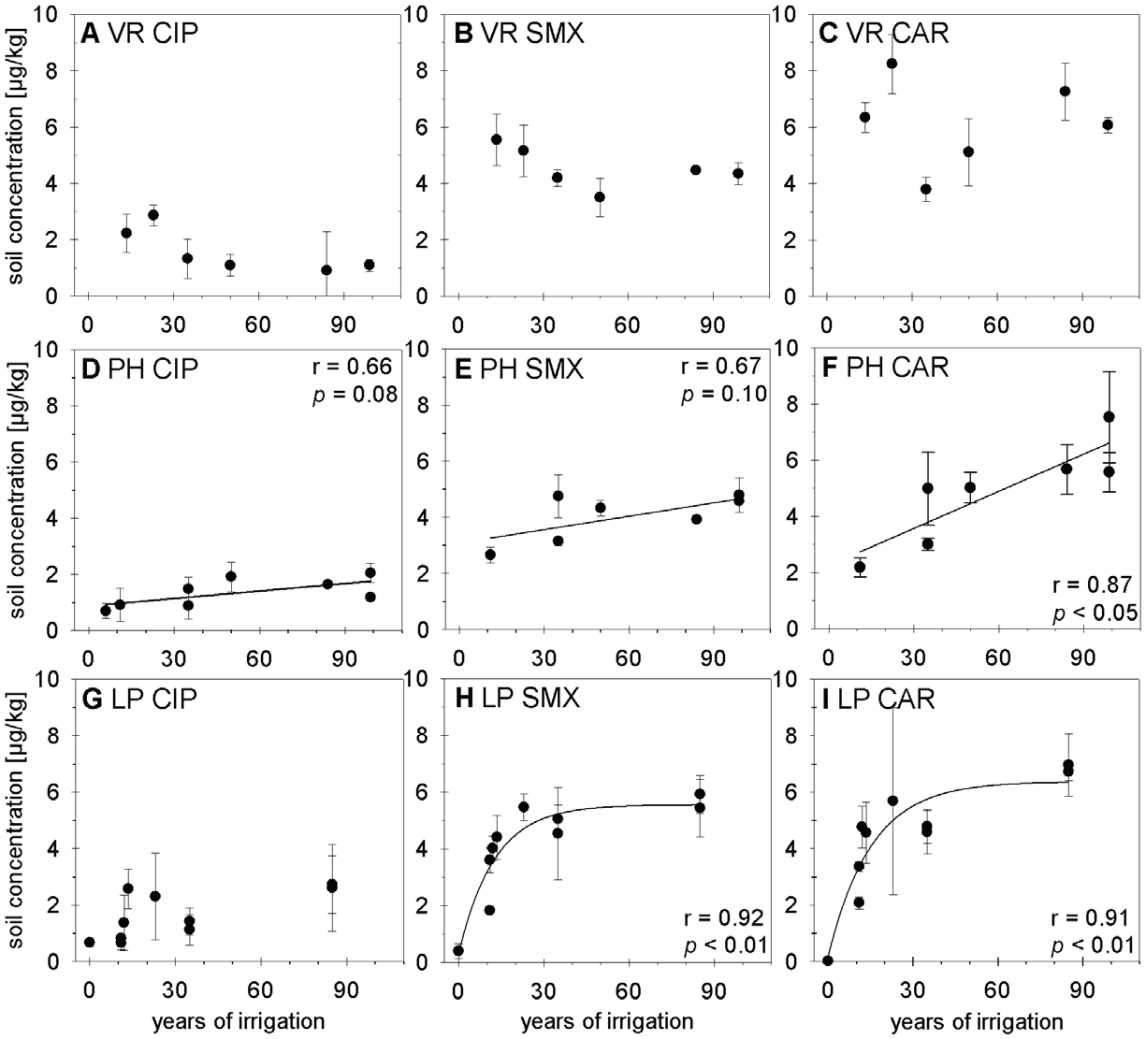
Concentrations of ciprofloxacin (CIP), sulfamethoxazole (SMX), and carbamazepine (CAR) in different soil types. Soils are classified as Vertisols (VR; panel A–C), Phaeozems (PH; panel D–F), and Leptosols (LP; panel G–I). Soils were irrigated repeatedly with untreated wastewater for different numbers of years; error bars indicate the standard deviation of concentrations in four quadrant parcels of individual fields.

Differences in the accumulation of compounds were not only observed between the pharmaceuticals but also between the different soil types. Total extracted concentrations of pharmaceuticals in Vertisols did not increase with irrigation time (Figure 2A–2C). This is at least partly due to the fact that Vertisols under rain-fed agriculture or with very short irrigation history were not analyzed because they are rare and we thus did not find such in the Mezquital Valley. In contrast, accumulation of sulfamethoxazole and carbamazepine in Leptosols followed a saturation function until a “steady-state” was approached (defined here as 95% of final concentration). Different from Leptosols, Phaeozems were characterized by a slower and more linear accumulation of ciprofloxacin, sulfamethoxazole, and carbamazepine (Figure 2D–2F). In comparison to Leptosols, Phaeozems are characterized by a thicker solum, resulting in higher fertility and productivity and therefore higher biological activity. These soil specific differences deserve closer inspection in future studies.

From an ecotoxicological point of view, it is crucial whether the observed increase in total contents of certain pharmaceuticals is related to the build-up of a bioaccessible and therefore potentially bioeffective pool of the respective agents. Calcium chloride-extractable concentrations of most agents that are deemed bioaccessible were usually smaller than 1.2 µg/kg (Figure S1A, S1C, S1E; Table S7). Maximum bioaccessible sulfamethoxazole concentrations equaled only one tenth of the respective total concentrations, and there was no increase of these concentrations over time, likely because efficient degradation [67] prevented the accumulation of easily accessible sulfamethoxazole. Only for the more recalcitrant compound carbamazepine a significant accumulation of CaCl_2_-extractable concentrations could be identified (Figure S1E).

An important question is whether the accumulation of pharmaceuticals, particularly of the antibiotic agents sulfamethoxazole and ciprofloxacin, affects the abundance of related *sul* and *qnr* resistance genes in the soils.

### Antibiotic Resistance Genes and Enterococci

**Figure 3:**
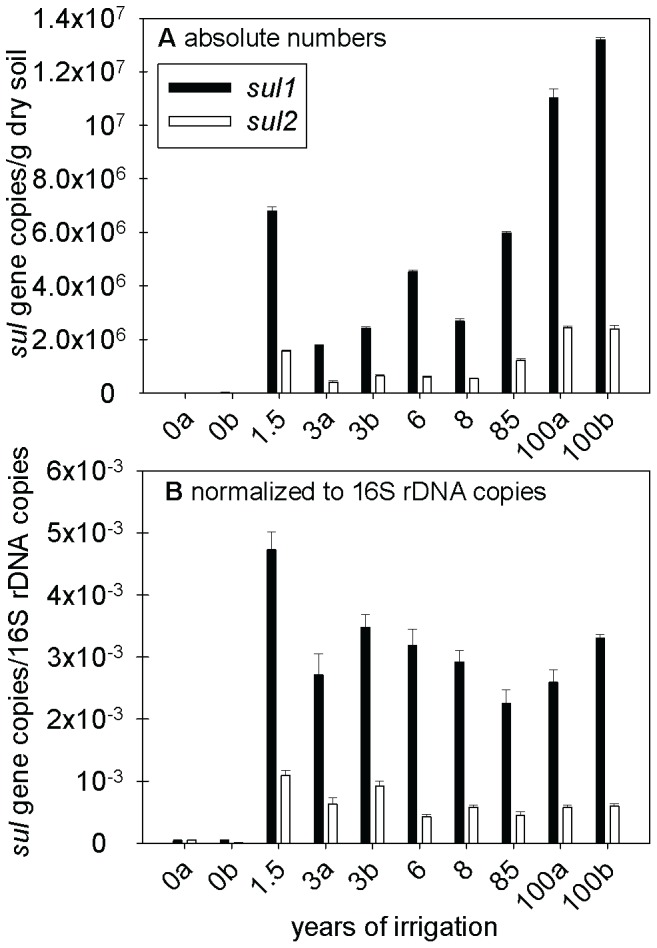
Absolute (panel A) and relative abundance (panel B) of *sul1*, *sul2* resistance genes in soils. Soils were irrigated repeatedly with untreated wastewater for different numbers of years. Error bars indicate the standard deviation between laboratory replications (three replicates) of the same composite soil sample for one field. The small letters “a” and “b” differentiate between fields that have been irrigated for the same period of time.

The *sul1* and *sul2* genes were present in all soils, even in those under rain-fed agriculture. Possible explanations are the presence of these genes in the “native” resistome of these soils, deposition of aerosols from wastewater-channels, or transport of resistant bacteria via dust or direct fertilization with human or animal excrements [41] or both. Absolute copy numbers of *sul1* resistance genes per g soil (DM) in irrigated soils exceeded those in soils under rain-fed agriculture by a factor of approximately 150–1500 (Figure 3A; exact numbers of resistance genes are provided in Tables S8 in the SI). There was a significant correlation between the absolute concentration of *sul1* genes and irrigation time (rank correlation coefficients: Kendalls tau = 0.69, p<0.05; Spearmans R = 0.80, p<0.05). The abundance of *sul2* in irrigated soils was 50–520 times larger than in soils without wastewater irrigation and also correlated with the duration of irrigation (Kendalls tau = 0.60 p<0.05; Spearmans R = 0.75, p<0.05). Hence, and unlike the accumulation of the detected sulfonamide, the abundance of the *sul* resistance genes continued to increase with increasing time of irrigation.

The absolute concentration of *sul1* genes we found in soils under rain-fed agriculture was still considerably smaller than 2.25×10^4^ to 7.57×10^4^ genes/g (DM) that Munir and Xagoraraki [40] reported for “background soil” before the application of manure or biosolids. Following the application of manure or biosolids, the copy numbers of *sul1* genes in their Michigan soils increased by 36% (manure) or 14% (biosolids) compared to background soils, which constitutes a much smaller increase than we observed following wastewater irrigation.

**Figure 4:**
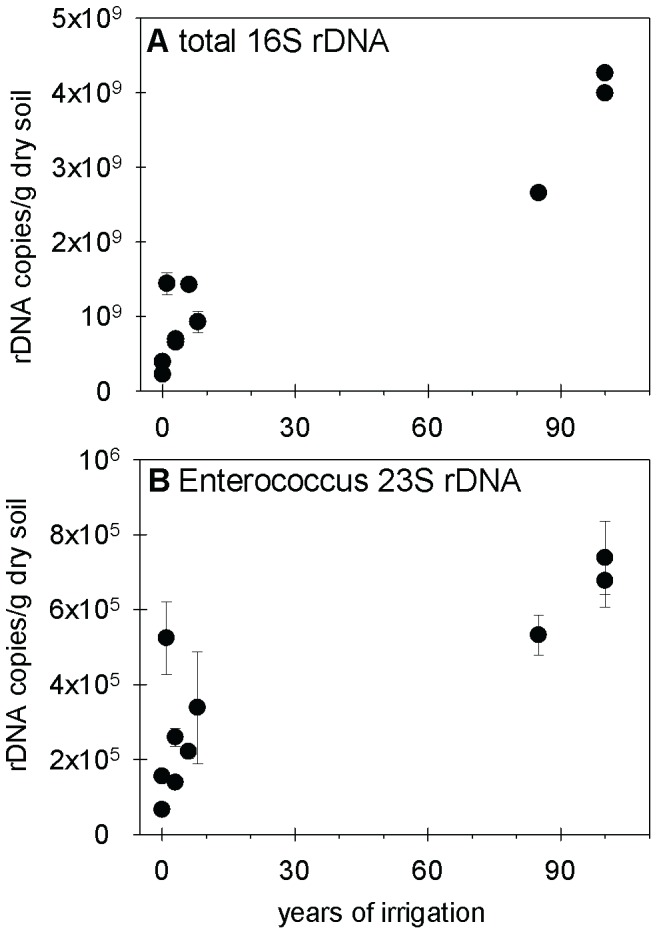
Gene copy numbers of total 16S rDNA (panel A) and *Enterococcus* spp. (panel B) in soils. Soils were irrigated repeatedly with untreated wastewater for different numbers of years. Error bars indicate the standard deviation between laboratory replications (three replicates) of the same soil sample.

Large absolute numbers of *sul* resistance genes in soils irrigated with wastewater for prolonged periods of time might be related to large contents of microbial biomass [72] and better survival of wastewater-derived bacteria in soil between irrigation events. To investigate the effect of wastewater-irrigation on the soil bacterial population we determined the total concentration of bacterial DNA by quantitative 16S rDNA PCR. The irrigated soils contained on average seven times more 16S rDNA than soils under rain-fed agriculture, and there was a significant correlation between irrigation time and 16S rDNA concentration in soil (Figure 4A; Kendalls tau = 0.74, p<0.05; Spearmans R = 0.86, p<0.05; exact numbers of 16S rDNA concentrations are provided in Table S9 in the SI). This increase of the total bacterial concentration due to wastewater-irrigation might at least partly be caused by the regular input of bacteria into the soil with wastewater. On the basis of 23S rDNA qPCR, we detected *Enterococcus* spp. in all soils, including soils with rain-fed agriculture. On average, the irrigated soils contained five times more *Enterococcus* genes than non-irrigated soils, and also the concentration of these genes was significantly correlated with the duration of irrigation (Figure 4B; Kendalls tau = 0.69, p<0.05; Spearmans R = 0.81, p<0.05; exact numbers of *Enterococcus* spp. are provided in Table S9 in the SI).

If the presence of pharmaceuticals, especially the accumulation of sulfamethoxazole and ciprofloxacin, in irrigated soils exerted a selective pressure on soil-dwelling microorganisms, then this should have increased the fraction of these organisms carrying the related *sul* and *qnr* resistance genes. This fraction of organisms carrying the respective gene can be expressed by normalizing the number of resistance genes to the concentration of total 16S rDNA. The irrigated soils were characterized by almost two orders of magnitude larger relative concentrations of *sul1* genes than the non-irrigated ones, and the relative concentrations of *sul2* genes in irrigated soils still exceeded the concentrations in non-irrigated soils on average by a factor of 22 (Figure 3B). However, different from the absolute concentrations discussed above, relative concentrations of *sul* genes were not correlated with the duration of irrigation: Soils irrigated with wastewater for 100 years did not contain more *sul* resistance genes in relation to their content of 16S rDNA than did a soil irrigated with wastewater for 1.5 years (Figure 3B; exact numbers of resistance genes are provided in Table S10 in the SI).

The relative abundance of *sul1* genes in wastewater-irrigated soils was comparable to the relative gene abundance reported by Heuer et al. [38] for a sandy soil treated with manure that contained the sulfonamide antibiotic sulfadiazine, but it was more than two orders of magnitude larger than the relative abundance reported for manure- or biosolid-amended soils by Munir and Xagoraki [40]. The relative abundance of *sul2* in the Mexican wastewater-irrigated soils is, however, much smaller than relative abundances of Heuer et al. [38]. Another important difference between the results of Heuer et al. [38] and our results is that repeated applications of sulfadiazine-containing manure caused a successive increase of the relative abundance of *sul* genes while no such increase was observed after prolonged irrigation with wastewater. Although the direct comparison of results is hampered by the different time-scales that were investigated (193 days in the study of Heuer et al. [38] versus multiple years of irrigation in our study), this difference can probably be related to different concentrations of bioaccessible sulfonamide antibiotics in these studies. The bioaccessible sulfadiazine concentrations in the experiment of Heuer et al [38] exceeded sulfamethoxazole concentrations in the Mezquital Valley soils by more than three orders of magnitude and also increased after repeated manure application, while no such increase of bioaccessible sulfamethoxazole concentrations with increasing irrigation time was observed for the Mexican soils. In another study of Heuer et al. [36], effects of sulfadiazine on the relative abundance of *sul2* decreased markedly when concentrations dropped below 150 µg/kg soil which is still approximately a factor of 15–30 larger than the concentrations of CaCl_2_-extractable sulfamethoxazole encountered in the Mezquital Valley soils. The bioaccessible sulfamethoxazole concentration in the soils of the Mezquital Valley thus is probably too small to induce a long-term accumulation of *sul* genes with increasing time of irrigation. Additionally, the accumulative, sequestered fraction of sulfamethoxazole most likely has no immediate impact on the abundance of *sul* genes, at least not in the concentration range present in this study.

No *qnrA* genes encoding fluoroquinolone resistance could be detected in any of the soils. Only *qnrB* and *qnrS* were found in two of the chronosequence soils (irrigated for six and 100 years) (Table S8 and Table S10 in the SI). Cummings et al. [37] detected five different quinolone resistance genes, *qnrA*, *qnrB*, and *qnrS* amongst others, in surface sediments from a sewage-impacted coastal wetland along the U.S.-Mexico border. Sediments of a nearby urban wetland that was largely unaffected by sewage contained (like the wastewater-irrigated soils) only three different *qnr* genes, amongst them *qnrB* and *qnrS*. Nucleotide sequences of cloned *qnrA* amplicons from the sediment were all similar to *qnrA* genes encoded on plasmids of clinical isolates, with only one exception. This differs from the present study in which no clinical *qnrA* genes were detected in the wastewater-irrigated soils of the Mezquital Valley. Although Gullberg et al. [42] showed that ciprofloxacin concentrations of less than 2.5 µg/L can cause selection of resistance in *in vitro* experiments, increasing total extractable concentrations of ciprofloxacin in soil were not correlated with increasing concentrations of *qnr* resistance genes. One reason for this might be the strong binding and hence small CaCl_2_-extractable concentrations of ciprofloxacin that also did not increase with time of irrigation. These results correspond to recent findings of Rosendahl et al. [57], suggesting that although the fluoroquinolone difloxacin is persistent in soil, its bioaccessible concentrations might be too small to affect microbial nitrogen transformation.

### Conclusions

Long-term irrigation of soils with untreated wastewater in the Mezquital Valley led to an accumulation of sulfamethoxazole, ciprofloxacin, and carbamazepine. This accumulation proceeded for a few decades, until after 19 to 28 years an upper limit was approached. This upper limit reflected steady-state conditions between pharmaceutical input and dissipation, but might also have been affected by the history of emissions with wastewater in the case of ciprofloxacin. The (bioaccessible) CaCl_2_-extractable concentrations of all compounds remained smaller than 1.2 µg/kg soil. Accordingly, the accumulation of sulfamethoxazole and ciprofloxacin in soil with increasing duration of irrigation was not accompanied by an increase of relative abundances of *sul* and *qnr* resistance genes. Nevertheless, the regular input of wastewater increased the relative concentrations of resistance genes in irrigated soils relative to soils under rain-fed agriculture. Furthermore, absolute concentrations of *sul1* resistance genes increased with increasing duration of irrigation, probably as a consequence of increasing microbial biomass and better survival of wastewater-derived bacteria between irrigation events after long-term irrigation.

## Supporting Information

Figure S1 **CaCl_2_-extractable and ASE-extractable concentrations in soils irrigated repeatedly with untreated wastewater for different numbers of years.** Error bars indicate the standard deviation of concentrations in four quadrant parcels of individual fields.
(TIF)

Table S1 **List of sampled sites.**
(DOC)

Table S2 **Recoveries of pharmaceuticals during ASE- and SPE-extractions.**
(DOC)

Table S3 **Extraction parameters of the Accelerated Solvent Extraction (ASE).**
(DOC)

Table S4 **Primers and probes for PCR and qPCR.**
(DOC)

Table S5 **Reagents and programs for qPCR.**
(DOC)

Table S6 **Total extracted pharmaceutical concentrations from soils irrigated repeatedly for different numbers of years with wastewater, standard deviation in brackets.**
(DOC)

Table S7 **CaCl_2_ extracted pharmaceutical concentrations from soils irrigated repeatedly for different numbers of years with wastewater, standard deviation in brackets.**
(DOC)

Table S8 **Concentrations of antibiotic resistance genes (average of gene copies and STD).**
(DOC)

Table S9 **Concentrations of** ***Enterococcus*** **spp. and 16S rDNA (average of gene copies and STD).**
(DOC)

Table S10 **Relative abundance of antibiotic resistance genes (average values and STD).**
(DOC)

Text S1 **Extraction of pharmaceuticals from soils.**
(DOC)

Text S2 **LC-MS/MS analysis.**
(DOC)

Text S3 **Quantification of antibiotic resistance genes and enterococci in soil samples.**
(DOC)
