# Correction: Accumulation of Pharmaceuticals, *Enterococcus*, and Resistance Genes in Soils Irrigated with Wastewater for Zero to 100 Years in Central Mexico

**DOI:** 10.1371/annotation/956a2a7c-714c-4233-ad3e-c7c0cefb1513

**Published:** 2012-12-05

**Authors:** Philipp Dalkmann, Melanie Broszat, Christina Siebe, Elisha Willaschek, Tuerkan Sakinc, Johannes Huebner, Wulf Amelung, Elisabeth Grohmann, Jan Siemens

There were errors in the Funding section. The correct funding information is as follows: This work was supported by grants (SI1106/5-1 and GR1792/4-1) from the German Research Foundation (DFG; http://www.dfg.de), and the Mexican Consejo Nacional de Sciencia y Technologia (CONACYT) under grant numbers CB 83767 and I 0110-193-10. The funders had no role in study design, data collection and analysis, decision to publish, or preparation of the manuscript. 

