# Supplementary material for: Accumulation of Pharmaceuticals, Enterococcus, and Resistance Genes in Soils Irrigated with Wastewater for Zero to 100 Years in Central Mexico
Source: PLoS One. 2012 Sep 25;7(9):e45397. doi: 10.1371/journal.pone.0045397 (PMC3458031; doi:10.1371/journal.pone.0045397)
Supplement: Table S1 — List of sampled sites. (DOC) [file pone.0045397.s002.doc]

**Table S1**: List of samples

| Sample-ID | Years of irrigation | Sampling date | Parameters analyzed | soil type |
| --- | --- | --- | --- | --- |
| 73-76 | 0 | 2009/08/20 | c^a^ | Leptosol |
| 97 | 0 | 2011/03/11 | c, m^b^ | n.d. |
| 98 | 0 | 2011/03/11 | c, m | n.d. |
| 119 | 0 | 2011/03/13 | c, m | n.d. |
| 115-118 | 1.5 | 2011/03/13 | c, m | n.d. |
| 93-96 | 3 | 2011/03/11 | c, m | n.d. |
| 99-102 | 3 | 2011/03/12 | c, m | n.d. |
| 103-106 | 6 | 2011/03/12 | c, m | Phaeozem |
| 111-114 | 8 | 2011/03/13 | c, m | n.d. |
| 69-72 | 11 | 2009/08/14 | c | Leptosol |
| 77-80 | 11 | 2009/08/24 | c | Leptosol |
| 85-88 | 11 | 2009/08/25 | c | Phaeozem |
| 13-16 | 12 | 2009/06/05 | c | n.d. |
| 81-84 | 13.5 | 2009/08/24 | c | Leptosol |
| 89-92 | 13.5 | 2009/08/25 | c | Vertisol |
| 61-64 | 23 | 2009/08/14 | c | Vertisol |
| 65-68 | 23 | 2009/08/14 | c | Leptosol |
| 01-04 | 35 | 2009/05/28 | c, m | Vertisol |
| 05-08 | 35 | 2009/05/29 | c | Phaeozem |
| 09-12 | 35 | 2009/05/29 | c | Leptosol |
| 17-20 | 35 | 2009/07/30 | c | n.d. |
| 57-60 | 35 | 2009/08/02 | c | Phaeozem |
| 45-48 | 50 | 2009/08/02 | c | Vertisol |
| 49-52 | 50 | 2009/08/02 | c | Phaeozem |
| 21-24 | 85 | 2009/07/30 | c | Leptosol |
| 25-28 | 85 | 2009/07/31 | c | Leptosol |
| 29-32 | 85 | 2009/07/31 | c | Vertisol |
| 33-36 | 85 | 2009/07/31 | c | Phaeozem |
| 107-110 | 85 | 2011/03/13 | c, m | n.d. |
| 37-40 | 100 | 2009/08/01 | c | Phaeozem |
| 41-44 | 100 | 2009/08/01 | c, m | Vertisol |
| 53-56 | 100 | 2009/08/02 | c | Phaeozem |

^a^ chemical analysis; ^m^ microbiological analysis, n.d.: not determined
