# Supplementary material for: Accumulation of Pharmaceuticals, Enterococcus, and Resistance Genes in Soils Irrigated with Wastewater for Zero to 100 Years in Central Mexico
Source: PLoS One. 2012 Sep 25;7(9):e45397. doi: 10.1371/journal.pone.0045397 (PMC3458031; doi:10.1371/journal.pone.0045397)
Supplement: Table S2 — Recoveries of pharmaceuticals during ASE- and SPE-extractions. (DOC) [file pone.0045397.s003.doc]

**Table S2:** Recoveries of ASE- and SPE-extractions

| Extraction | Ciprofloxacin | Enrofloxacin | Sulfamethoxazole | Trimethoprim | Clarithromycin | Carbamazepine | Naproxen | Diclofenac | Bezafibrate |
| --- | --- | --- | --- | --- | --- | --- | --- | --- | --- |
|  | [%] | [%] | [%] | [%] | [%] | [%] | [%] | [%] | [%] |
| Accelerated Solvent Extraction (ASE) | 89 | 78 | 54 | 83 | 93 | 77 | 95 | 85 | 96 |
| Solid Phase Extraction (SPE) |  |  |  |  |  |  |  |  |  |
| Oasis HLB | 74 | 76 | 84 | 32 | 99 | 97 | 90 | 72 | 105 |
| Chromabond SB | 0.0 | 0.0 | 0.0 | 0.4 | 0.0 | 0.2 | 0.0 | 0.0 | 0.0 |
