# Supplementary material for: Accumulation of Pharmaceuticals, Enterococcus, and Resistance Genes in Soils Irrigated with Wastewater for Zero to 100 Years in Central Mexico
Source: PLoS One. 2012 Sep 25;7(9):e45397. doi: 10.1371/journal.pone.0045397 (PMC3458031; doi:10.1371/journal.pone.0045397)
Supplement: Table S3 — Extraction parameters of the Accelerated Solvent Extraction (ASE). (DOC) [file pone.0045397.s004.doc]

Table S3: Extraction parameters of the Accelerated Solvent Extraction (ASE)

| Parameter | Method 1 (after Gobel et al.^a^) | Method 2 (after Golet et al.^b^) |
| --- | --- | --- |
| Solvent | methanol : Millipore-water  (1:1) (v/v) | 50 mM aqueous H_3_PO_4_ : acetonitrile  (1:1) (v/v) |
| Pressure [bar] | 100 | 100 |
| Temperature [°C] | 100 | 100 |
| Heating time [min] | 5 | 5 |
| Static extraction [min] | 5 | 10 |
| Extraction cycles | 2 | 3 |
| Flush volume [%] | 60 | 90 |
| Flush with N_2_ [sec] | 60 | 180 |

^a^ [1]; ^b^ [2]

**REFERENCES**

1. Gobel A, Thomsen A, McArdell CS, Alder AC, Giger W, et al. (2005) Extraction and determination of sulfonamides, macrolides, and trimethoprim in sewage sludge. Journal of Chromatography A 1085: 179-189.

2. Golet EM, Strehler A, Alder AC, Giger W (2002) Determination of fluoroquinolone antibacterial agents in sewage sludge and sludge-treated soil using accelerated solvent extraction followed by solid-phase extraction. Analytical Chemistry 74: 5455-5462.
