# Supplementary material for: Accumulation of Pharmaceuticals, Enterococcus, and Resistance Genes in Soils Irrigated with Wastewater for Zero to 100 Years in Central Mexico
Source: PLoS One. 2012 Sep 25;7(9):e45397. doi: 10.1371/journal.pone.0045397 (PMC3458031; doi:10.1371/journal.pone.0045397)
Supplement: Table S4 — Primers and probes for PCR and qPCR. (DOC) [file pone.0045397.s005.doc]

Table S4: Primers and probes for PCR and qPCR

| Target | Amplicon size bp | Oligonucleotide/TaqMan probe | Sequence (5´ to 3´) | T_a_  (°C) | Reference |
| --- | --- | --- | --- | --- | --- |
| Bacteria | 439 | Bact348f | AGGCAGCAGTDRGGAAT | 50 | [1] |
| 16S rDNA |  | Bact786r | GGACTACYVGGGTATCTAAT |  |  |
|  |  | tpBact | TGCCAGCAGCCGCGGTAATACRDAG |  |  |
| *sul1* | 67 | qSUL653F | CCGTTGGCCTTCCTGTAAAG | 58 | [2] |
|  |  | qSUL719R | TTGCCGATCGCGTGAAGT |  |  |
|  |  | tpSUL1 | CAGCGAGCCTTGCGGCGG |  |  |
| *sul2* | 60 | qsul-595F | CGGCTGCGCTTCGATTT | 51 | [3] |
|  |  | qsul2-654R | CGCGCGCAGAAAGGATT |  |  |
|  |  | tpsul2-614 | CGGTGCTTCTGTCTGTTTCGCGC |  |  |
| *qnrA* | 543 | qnrA-F | GATAAAGTTTTTCAGCAAGAGG | 56 | [4] |
|  |  | qnrA-R | ATCCAGATCGGCAAAGGTTA |  |
| *qnrB* | 497 | qnrB-F | AGCGGCACTGAATTTAT | 56 | [4] |
|  |  | qnrB-R | GTTTGCTGCTCGCCAGTC |  |  |
| *qnrS* | 600 | qnrS-F | GGAAACCTACAATCATACATA | 56 | [4] |
|  |  | qnrS-R | GTCAGGATAAACAACAATACC |  |  |
| *Enterococcus* spp. | 70 | ECST784F | AGA AAT TCC AAA CGA ACT TG | 56 | [5] |
| 23S rDNA |  | ENC854R | CAG TGC TCT ACC TCC ATC ATT |  |  |
|  |  | GPL813TQ | TGG TTC TCT CCG AAA TAG CTT TAG GGC TA |  |  |

T_a_: annealing temperature; D: G, A or T, R: A or G, Y: C or T, V: G, A or C

**REFERENCES**

1. Takai K, Horikoshi K (2000) Rapid detection and quantification of members of the archaeal community by quantitative PCR using fluorogenic probes. Applied and Environmental Microbiology 66: 5066-5072.

2. Heuer H, Smalla K (2007) Manure and sulfadiazine synergistically increased bacterial antibiotic resistance in soil over at least two months. Environmental Microbiology 9: 657-666.

3. Heuer H, Focks A, Lamshoeft M, Smalla K, Matthies M, et al. (2008) Fate of sulfadiazine administered to pigs and its quantitative effect on the dynamics of bacterial resistance genes in manure and manured soil. Soil Biology & Biochemistry 40: 1892-1900.

4. Guillard T, Cavallo JD, Cambau E, Duval V, Bajolet O, et al. (2010) Real-time PCR for fast detection of plasmid-mediated *qnr* genes in extended spectrum beta-lactamase producing Enterobacteriaceae. Pathologie Biologie 58: 430-433.

5. Frahm E, Obst U (2003) Application of the fluorogenic probe technique (TaqMan PCR) to the detection of *Enterococcus* spp. and *Escherichia coli* in water samples. Journal of Microbiological Methods 52: 123-131.
