# Supplementary material for: Accumulation of Pharmaceuticals, Enterococcus, and Resistance Genes in Soils Irrigated with Wastewater for Zero to 100 Years in Central Mexico
Source: PLoS One. 2012 Sep 25;7(9):e45397. doi: 10.1371/journal.pone.0045397 (PMC3458031; doi:10.1371/journal.pone.0045397)
Supplement: Table S5 — Reagents and programs for qPCR. (DOC) [file pone.0045397.s006.doc]

**Table** **S5:** Reagents and programs for qPCR

| Assay | Reagents (for 10 µL final volume) | Program |
| --- | --- | --- |
| 16S rDNA | 2 µl LightCycler®TaqMan Master (Roche Diagnostics, Mannheim, Germany), 0.5 µM of each primer, 0.15 µM TaqMan probe and 0.875 mM MgCl_2_ | 95°C for 10 min  45 cycles of 95°C 10 s, 56°C 5 s, 72°C 20 s |
| *sul1* | 2 µl LightCycler®TaqMan Master (Roche Diagnostics, Mannheim, Germany), 0.4 µM of each primer, 0.15 µM TaqMan probe and  0.5  mM MgCl_2_ | 95°C for 10 min  45 cycles of 95°C 10 s, 58°C 30 s, 72°C 1 s |
| *sul2* | 2 µl LightCycler®TaqMan Master (Roche Diagnostics, Mannheim, Germany), 0.7 µM of each primer, 0.15 µM TaqMan probe and  0.75 mM MgCl_2_ | 95°C for 10 min  45 cycles of 95°C 10 s, 51°C 40 s, 72°C 1 s |
| *qnrA* | 1 µl LightCycler® FastStart DNA Master SYBR Green I (Roche Diagnostics, Mannheim, Germany), 0.4 µM of each primer and 4 mM MgCl_2_ | 95°C for 10 min  45 cycles of 95°C 10 s, 51°C 40 s, 72°C 1 s  Melting curves:  10 s 95°C, continuous heating at 0.1°C/s from 65°C to 95°C |
| *qnrB* and *qnrS* | 1 µl LightCycler® FastStart DNA Master SYBR Green I (Roche Diagnostics, Mannheim, Germany), 0.5 µM of each primer and 4 mM MgCl_2_ | 95°C for 10 min  45 cycles of 95°C 10 s, 51°C 40 s, 72°C 1 s  Melting curves:  10 s 95°C, continuous heating at 0.1°C/s from 65°C to 95°C |
| *Enterococcus* spp. | 2 µl LightCycler®TaqMan Master (Roche Diagnostics, Mannheim, Germany), 0.4 µM of each primer, 0.15 µM TaqMan probe and  0.5 mM MgCl_2_ | 95°C for 10 min  45 cycles of 95°C 10 s, 56°C 30 s, 72°C 1 s |
