# Supplementary material for: Accumulation of Pharmaceuticals, Enterococcus, and Resistance Genes in Soils Irrigated with Wastewater for Zero to 100 Years in Central Mexico
Source: PLoS One. 2012 Sep 25;7(9):e45397. doi: 10.1371/journal.pone.0045397 (PMC3458031; doi:10.1371/journal.pone.0045397)
Supplement: Table S6 — Total extracted pharmaceutical concentrations from soils irrigated repeatedly for different numbers of years with wastewater, standard deviation in brackets. (DOC) [file pone.0045397.s007.doc]

**Table S6:** Total extracted pharmaceutical concentrations from soils irrigated repeatedly for different numbers of years with wastewater, standard deviation in brackets

| Compound | Soil concentration [µg/kg] | | | | | | | | | | | | |
| --- | --- | --- | --- | --- | --- | --- | --- | --- | --- | --- | --- | --- | --- |
|  | 0 years | 1.5 years | 3 years | 6 years | 8 years | 11 years | 12 years | 13.5 years | 23 years | 35 years | 50 years | 85 years | 100 years |
| ciprofloxacin | 0.67 (0.37) | 0.42 | 0.79 (0.65) | 0.68 | 0.35 | 0.79 (0.13) | 1.37 | 2.40 (0.24) | 2.58 (0.40) | 1.24 (0.24) | 1.50 (0.58) | 2.82 (0.82) | 1.44 (0.53) |
| enrofloxacin | 0.03 (0.07) | 0.14 | 0.26 (0.27) | 0.15 | 0.20 | 0.18 (0.02) | 0.03 | 0.16 (0.09) | 0.22 (0.12) | 0.38 (0.30) | 0.57 (0.21) | 0.39 (0.41) | 0.64 (0.54) |
| sulfamethoxazole | 0.11 (0.19) | 2.39 | 2.34 | 3.01 | 3.03 | 2.69 (0.89) | 4.02 | 4.98 (0.80) | 5.31 (0.22) | 4.33 (0.73) | 3.91 (0.58) | 5.14 (0.91) | 4.57 (0.22) |
| trimethoprim | 0.14 (0.23) | 0.79 | 0.23 (0.21) | 0.45 | 0.75 | 0.76 (0.15) | 1.32 | 2.36 (0.23) | 2.39 (0.29) | 1.05 (0.21) | 1.32 (0.56) | 2.16 (0.69) | 1.57 (0.47) |
| clarithromycin | 0.55 (1.10) | n.m.^a^ | n.m. | n.m. | n.m. | 1.87 (0.92) | 0.68 | 1.82 (0.13) | 3.89 (2.19) | 1.65 (0.79) | 0.87 (0.12) | 1.34 (0.71) | 2.44 (1.18) |
| carbamazepine | 0.02 (0.02) | 1.49 | 2.03 (0.10) | 3.61 | 2.54 | 2.54 (0.71) | 4.76 | 5.45 (1.25) | 6.96 (1.80) | 4.23 (0.82) | 5.06 (0.07) | 6.76 (0.64) | 6.39 (1.02) |
| naproxen | 0.56 (0.70) | 2.86 | 1.48 (0.89) | 2.75 | 2.83 | 0.99 (0.40) | 1.36 | 1.55 (0.55) | 1.65 (0.36) | 1.68 (0.49) | 0.76 (0.23) | 1.89 (0.83) | 1.09 (0.31) |
| diclofenac | 0.05 (0.06) | 0.40 | 0.13 (0.09) | 0.34 | 0.20 | 0.31 (0.21) | 0.20 | 0.18 (0.03) | 0.32 (0.04) | 0.12 (0.03) | 0.14 (0.00) | 0.23 (0.13) | 0.20 (0.05) |
| bezafibrate | 0.01 (0.02) | 0.67 | n.m. | 0.52 | 0.30 | 0.18 (0.09) | 0.35 | 0.29 (0.04) | 0.61 (0.05) | 0.12 (0.03) | 0.21 (0.05) | 0.42 (0.36) | 0.26 (0.10) |

^a^ not measured
