# Supplementary material for: Accumulation of Pharmaceuticals, Enterococcus, and Resistance Genes in Soils Irrigated with Wastewater for Zero to 100 Years in Central Mexico
Source: PLoS One. 2012 Sep 25;7(9):e45397. doi: 10.1371/journal.pone.0045397 (PMC3458031; doi:10.1371/journal.pone.0045397)
Supplement: Table S7 — CaCl2 extracted pharmaceutical concentrations from soils irrigated repeatedly for different numbers of years with wastewater, standard deviation in brackets. (DOC) [file pone.0045397.s008.doc]

**Table S7:** CaCl_2_ extracted pharmaceutical concentrations from soils irrigated repeatedly for different numbers of years with wastewater, standard deviation in brackets

| Compound | Soil concentration [µg/kg] | | | | | | | | | | | | |
| --- | --- | --- | --- | --- | --- | --- | --- | --- | --- | --- | --- | --- | --- |
|  | 0 years | 1.5 years | 3 years | 6 years | 8 years | 11 years | 12 years | 13.5 years | 23 years | 35 years | 50 years | 85 years | 100 years |
| ciprofloxacin | 0.07 (0.05) | 0.14 | 0.22 (0.04) | 0.11 | 0.12 | 0.15 (0.03) | 0.41 | 0.14 (0.02) | 0.35 (0.25) | 0.46 (0.04) | 0.65 (0.24) | 0.55 (0.39) | 0.70 (0.44) |
| enrofloxacin | 0.02 (0.01) | 0.03 | 0.13 (0.07) | 0.04 | 0.06 | 0.06 (0.03) | 0.04 | 0.02 (0.00) | 0.13 (0.07) | 0.12 (0.11) | 0.34 (0.09) | 0.40 (0.53) | 0.42 (0.44) |
| sulfamethoxazole | 0.02 (0.04) | 0.36 | 0.19 | 0.28 | 0.20 | 0.24 (0.18) | 0.30 | 0.40 (0.13) | 0.45 (0.31) | 0.33 (0.16) | 0.26 (0.04) | 0.23 (0.19) | 0.38 (0.15) |
| trimethoprim | 0.07 (0.05) | 0.02 | 0.01 (0.00) | 0.02 | 0.01 | 0.01 (0.01) | 0.01 | 0.01 (0.00) | 0.01 (0.00) | 0.01 (0.01) | 0.01 (0.00) | 0.01 (0.01) | 0.01 (0.00) |
| clarithromycin | 0.12 (0.09) | 0.10 | 0.09 (0.03) | 0.12 | 0.03 | 0.10 (0.10) | 0.00 | 0.10 (0.08) | 0.08 (0.11) | 0.00 (0.00) | 0.00 (0.00) | 0.01 (0.01) | 0.01 (0.00) |
| carbamazepine | 0.00 (0.00) | 0.40 | 0.20 (0.03) | 0.31 | 0.32 | 0.23 (0.11) | 0.31 | 0.40 (0.08) | 0.51 (0.03) | 0.43 (0.08) | 0.47 (0.13) | 0.49 (0.11) | 0.41 (0.09) |
| naproxen | 0.38 (0.25) | 0.96 | 0.46 (0.00) | 0.41 | 0.67 | 0.31 (0.14) | 0.41 | 0.63 (0.21) | 0.52 (0.35) | 0.47 (0.10) | 0.20 (0.15) | 0.50 (0.26) | 0.26 (0.11) |
| diclofenac | 0.00 (0.01) | 0.15 | 0.03 (0.00) | 0.04 | 0.01 | 0.02 (0.02) | 0.00 | 0.01 (0.00) | 0.01 (0.01) | 0.00 (0.00) | 0.00 (0.00) | 0.01 (0.01) | 0.00 (0.00) |
| bezafibrate | 0.00 (0.00) | 0.22 | 0.01 (0.01) | 0.04 | 0.02 | 0.01 (0.01) | 0.01 | 0.01 (0.00) | 0.02 (0.01) | 0.00 (0.00) | 0.01 (0.01) | 0.02 (0.04) | 0.01 (0.01) |
