# Supplementary material for: Accumulation of Pharmaceuticals, Enterococcus, and Resistance Genes in Soils Irrigated with Wastewater for Zero to 100 Years in Central Mexico
Source: PLoS One. 2012 Sep 25;7(9):e45397. doi: 10.1371/journal.pone.0045397 (PMC3458031; doi:10.1371/journal.pone.0045397)
Supplement: Table S8 — Concentrations of antibiotic resistance genes (average of gene copies and STD). (DOC) [file pone.0045397.s009.doc]

Table S8: Antibiotic resistance genes (average of gene copies and STD)

| Sample-ID | irrigation length [years] | *sul1*/g soil (DM) | *sul2*/g soil (DM) | *qnrA*/g soil (DM^a^) | *qnrB*/g soil (DM^a^) | *qnrS*/g soil (DM^a^) |
| --- | --- | --- | --- | --- | --- | --- |
| 97 | 0 a | (8.64 ± 2.69)×10^3^ | (1.06 ± 0.06)×10^4^ | n.d. | n.d. | n.d. |
| 98 | 0 b | (1.86 ± 0.18)×10^4^ | (4.56 ± 0.23)×10^3^ | n.d. | n.d. | n.d. |
| 115-118 | 1.5 | (6.80 ± 0.15)×10^6^ | (1.57 ± 0.04)×10^6^ | n.d. | n.d. | n.d. |
| 93-96 | 3 a | (1.77 ± 0.04)×10^6^ | (4.11 ± 0.40)×10^5^ | n.d. | n.d. | n.d. |
| 99-102 | 3 b | (2.41 ± 0.07)×10^6^ | (6.39 ± 0.36)×10^5^ | n.d. | n.d. | n.d. |
| 103-106 | 6 | (4.53 ± 0.06)×10^6^ | (6.07 ± 0.23)×10^5^ | n.d. | (7.40 ± 0.02)×10^4^ | (3.00 ± 4.18)×10^2^ |
| 111-114 | 8 | (2.69 ± 0.09)×10^6^ | (5.37 ± 0.17)×10^5^ | n.d. | n.d. | n.d. |
| 107-110 | 85 | (5.98 ± 0.06)×10^6^ | (1.21 ± 0.07)×10^6^ | n.d. | n.d. | n.d. |
| 120 | 100 a | (1.10 ± 0.03)×10^7^ | (2.45 ± 0.05)×10^6^ | n.d. | n.d. | (5.25 ± 4.42)×10^2^ |
| 121 | 100 b | (1.32 ± 0.01)×10^7^ | (2.39 ± 0.12)×10^6^ | n.d. | n.d. | n.d. |

^a^ dry matter; n.d.: not detectable
