# Supplementary material for: Accumulation of Pharmaceuticals, Enterococcus, and Resistance Genes in Soils Irrigated with Wastewater for Zero to 100 Years in Central Mexico
Source: PLoS One. 2012 Sep 25;7(9):e45397. doi: 10.1371/journal.pone.0045397 (PMC3458031; doi:10.1371/journal.pone.0045397)
Supplement: Table S9 — Concentrations of Enterococcus spp. and 16S rDNA (average of gene copies and STD). (DOC) [file pone.0045397.s010.doc]

**Table S9:** *Enterococcus* spp. and 16S rDNA (average of gene copies and STD)

| Sample-ID | irrigation length [years] | *Enterococcus* spp*.*/g soil (DM^a^) | 16S rDNA/g soil (DM^a^) |
| --- | --- | --- | --- |
| 97 | 0 a | (6.54 ± 0.53)×10^4^ | (2.20 ± 0.05)×10^8^ |
| 98 | 0 b | (1.55 ± 0.15)×10^5^ | (3.90 ± 0.18)×10^8^ |
| 115-118 | 1.5 | (5.23 ± 0.97)×10^5^ | (1.44 ± 0.15)×10^9^ |
| 93-96 | 3a | (2.59 ± 0.24)×10^5^ | (6.50 ± 0.23)×10^8^ |
| 99-102 | 3b | (1.39 ± 0.16)×10^5^ | (6.94 ± 0.15)×10^8^ |
| 103-106 | 6 | (2.21 ± 0.18)×10^5^ | (1.42 ± 0.02)×10^9^ |
| 111-114 | 8 | (3.38 ± 1.49)×10^5^ | (9.22 ± 1.44)×10^8^ |
| 107-110 | 85 | (5.31 ± 0.54)×10^5^ | (2.65 ± 0.05)×10^9^ |
| 120 | 100 a | (7.38 ± 0.98)×10^5^ | (4.26 ± 0.09)×10^9^ |
| 121 | 100 b | (6.77 ± 0.70)×10^5^ | (3.99 ± 0.06)×10^9^ |

^a^ dry matter
