# Supplementary material for: Accumulation of Pharmaceuticals, Enterococcus, and Resistance Genes in Soils Irrigated with Wastewater for Zero to 100 Years in Central Mexico
Source: PLoS One. 2012 Sep 25;7(9):e45397. doi: 10.1371/journal.pone.0045397 (PMC3458031; doi:10.1371/journal.pone.0045397)
Supplement: Table S10 — Relative abundance of antibiotic resistance genes (average values and STD). (DOC) [file pone.0045397.s011.doc]

**Table S10: Relative abundance of antibiotic resistance genes (average values and STD)**

| Sample-ID | irrigation length [years] | *sul1*/16S rDNA | *sul2*/16S rDNA | *qnrA*/16S rDNA | *qnrB*/16S rDNA | *qnrS*/16S rDNA |
| --- | --- | --- | --- | --- | --- | --- |
| 97 | 0 a | (3.93 ± 1.40)×10^-5^ | (4.82 ± 0.44)×10^-5^ | n.d.^a^ | n.d. | n.d. |
| 98 | 0 b | (4.76 ± 0.59)×10^-5^ | (1.17 ± 0.08)×10^-5^ | n.d. | n.d. | n.d. |
| 115-118 | 1.5 | (4.73 ± 0.29)×10^-3^ | (1.09 ± 0.08)×10^-3^ | n.d. | n.d. | n.d. |
| 93-96 | 3 a | (2.72 ± 0.34)×10^-3^ | (6.32 ± 1.06)×10^-4^ | n.d. | n.d. | n.d. |
| 99-102 | 3 b | (3.48 ± 0.21)×10^-3^ | (9.21 ± 0.78)×10^-4^ | n.d. | n.d. | n.d. |
| 103-106 | 6 | (3.19 ± 0.26)×10^-3^ | (4.27 ± 0.40)×10^-4^ | n.d. | (4.26 ± 1.67)×10^-5^ | (2.36 ± 2.92)×10^-7^ |
| 111-114 | 8 | (2.92 ± 0.18)×10^-3^ | (5.82 ± 0.36)×10^-4^ | n.d. | n.d. | n.d. |
| 107-110 | 85 | (2.25 ± 0.22)×10^-3^ | (4.55 ± 0.54)×10^-4^ | n.d. | n.d. | n.d. |
| 120 | 100 a | (2.59 ± 0.21)×10^-3^ | (5.74 ± 0.43)×10^-4^ | n.d. | n.d. | (1.67 ± 1.29)×10^-6^ |
| 121 | 100 b | (3.31 ± 0.06)×10^-3^ | (5.99 ± 0.41)×10^-4^ | n.d. | n.d. | n.d. |

^a^ not detectable
