# Supplementary material for: Accumulation of Pharmaceuticals, Enterococcus, and Resistance Genes in Soils Irrigated with Wastewater for Zero to 100 Years in Central Mexico
Source: PLoS One. 2012 Sep 25;7(9):e45397. doi: 10.1371/journal.pone.0045397 (PMC3458031; doi:10.1371/journal.pone.0045397)
Supplement: Text S1 — Extraction of pharmaceuticals from soils. (DOC) [file pone.0045397.s012.doc]

**Text S1:** Extraction of pharmaceuticals from soil samples

Soil samples were lyophilized and sieved to a grain size < 2 mm. We distributed ten grams of dry matter (DM) of each soil into borosilicate centrifuge glasses. The extraction of an easily extractable, “bioaccessible” compound fraction was performed with 25 mL of a 0.01 M CaCl_2_ solution. For this purpose, we added the CaCl_2_ solution to the soil aliquots which were afterwards shaken end-over-end in an overhead shaker for 24 h followed by centrifugation at 2500 × g for 20 min. Supernatants were decanted and acidified to a pH of 2.4 with 12 M hydrochloric acid (HCl). Afterwards, we added fifty microliter of the internal standard solution as cocktail with a concentration of 1 µg/mL of each isotope-labeled pharmaceutical. To pre-concentrate and purify the samples, we performed solid phase extractions (SPE) with Oasis HLB cartridges (Waters, Milford, MA/USA). The cartridges were conditioned with 5 mL of methanol and equilibrated with 5 mL of acidified Millipore water (HCl, pH 2.4). After the sample had passed, the cartridges were washed with 6 mL of acidified Millipore water (HCl, pH 2.4). Compounds were eluted from the dried cartridges with 4 mL methanol, 4 mL acetonitrile, and 4 mL acetonitrile acidified with 0.1% of a 12 M HCl. This solution was evaporated in a rotary evaporator almost to dryness. Subsequently, we added 1 mL of an aqueous 50 mM phosphoric acid:acetonitrile solution (80:20, v/v). After ultrasonification and ultracentrifugation (15000 × g, 20 min) we transferred the samples to HPLC vials and stored them at -21°C until measurement.

To assess the strongly bound, sequestered fraction of pharmaceuticals in soil, the CaCl_2_-extracted soil samples were lyophilized again and extracted via accelerated solvent extraction (ASE). We combined two different solvents for the extraction to account for the different physico-chemical properties of the pharmaceuticals (Table 2 in the main article). We used an aqueous 50 mM phosphoric acid:acetonitrile solution (50:50, v/v; according to Golet et al. [1] and a methanol:water solution (50:50, v/v; according to Gobel et al. [2]). Details on the ASE-extraction procedure are given in Table S3. Extraction recoveries of the extraction method varied between 54-95%. Recovery rates for the individual compounds are presented in Table S2. Following extraction, aliquots of 30 mL were diluted to a volume of 300 mL with Millipore water and acidified with HCl to a pH of 2.4. After adding 50 µL of the internal standard solution, solid phase extractions were conducted similar to the CaCl_2_ extracts, but with an additional strong anion exchange (SAX) cartridge (Chromabond SB; Macherey-Nagel, Düren/Germany) in front of the HLB cartridge for additional sample clean-up and to prevent the HLB cartridge from clogging. A recovery experiment proved negligible retention of the analyzed pharmaceuticals on the SAX cartridges (less than 0.5% of the target compounds were lost, Table S2). Recoveries from the Oasis HLB cartridges are also shown in Table S2. All chemicals were of HPLC gradient grade quality.

**REFERENCES**

1. Golet EM, Strehler A, Alder AC, Giger W (2002) Determination of fluoroquinolone antibacterial agents in sewage sludge and sludge-treated soil using accelerated solvent extraction followed by solid-phase extraction. Analytical Chemistry 74: 5455-5462.

2. Gobel A, Thomsen A, McArdell CS, Alder AC, Giger W, et al. (2005) Extraction and determination of sulfonamides, macrolides, and trimethoprim in sewage sludge. Journal of Chromatography A 1085: 179-189.
