# Supplementary material for: Accumulation of Pharmaceuticals, Enterococcus, and Resistance Genes in Soils Irrigated with Wastewater for Zero to 100 Years in Central Mexico
Source: PLoS One. 2012 Sep 25;7(9):e45397. doi: 10.1371/journal.pone.0045397 (PMC3458031; doi:10.1371/journal.pone.0045397)
Supplement: Text S2 — LC-MS/MS analysis. (DOC) [file pone.0045397.s013.doc]

**Text S2:** LC-MS/MS analysis

The analysis of pharmaceutical concentrations in soil extracts was performed with liquid chromatography tandem mass spectrometry (LC-MS/MS). A ThermoFinnigan system was used that was composed of a Surveyor autosampler plus, a Surveyor MS pump plus, and a TSQ Quantum Ultra tandem mass spectrometer equipped with an heated electrospray ionization ion source (HESI) operating in positive mode (Thermo Finnigan, Dreieich, Germany). The separation of pharmaceuticals was achieved with an XBridge C18 3.5 µm, 2.1x150 mm (Waters, Milford, MA, USA) HPLC column with guard column (Sentry 2.1x10 mm, Waters, Milford, MA, USA). All pharmaceuticals were analyzed in the same run. Eluents were methanol (A) and Millipore water (B) both acidified with 0.1% formic acid. The flow rate was 300 µL/min. The gradient elution started with 5% A, increasing after 5 min to 60%, raising from 60 to 80% after 15 min, further raising from 80 to 95% after 16 min, maintaining 95% for 0.5 min, and then back to initial conditions in 0.5 min. These initial conditions were kept until method end after 25 min. For CaCl_2_- and ASE- extracts, 10 µL of sample were injected into the system using partial loop injection. Ionization parameters were: discharge current 4.0 kV, vaporizer temperature 390°C, and capillary temperature 217°C. Nitrogen served as sheath and auxiliary gas and helium was used as collision gas at a pressure of 1.5 mTorr. The MS was operated in selected reaction monitoring (SRM) mode with at least two transitions being measured for each compound.
