# Supplementary material for: Accumulation of Pharmaceuticals, Enterococcus, and Resistance Genes in Soils Irrigated with Wastewater for Zero to 100 Years in Central Mexico
Source: PLoS One. 2012 Sep 25;7(9):e45397. doi: 10.1371/journal.pone.0045397 (PMC3458031; doi:10.1371/journal.pone.0045397)
Supplement: Text S3 — Quantification of antibiotic resistance genes and enterococci in soil samples. (DOC) [file pone.0045397.s014.doc]

**Text S3**: Quantification of antibiotic resistance genes and enterococci in soil samples

Aliquots of soil samples from fields of the chronosequence irrigated for 0, 1.5, 3, 6, 8, 85, and 100 years were analyzed by real-time qPCR, in the following denominated as qPCR. We did not analyze soil samples of other irrigation durations specified above, because these samples were transported with 24 h delay from Mexico to Germany by the airline and were partly thawed by arrival. For each composite sample we pooled 10 g of the four parcels. Total DNA was extracted from 500 mg soil using the NucleoSpin® Soil kit according to the manufacturer’s protocol (Macherey-Nagel, Düren, Germany) and eluted with 50 µL elution buffer. Prior to qPCR we diluted the DNA solutions 1:5 (1:50 for 16S rDNA) with Tris-EDTA buffer (10 mM Tris, 1 mM EDTA, pH 8) or Tris-EDTA buffer containing 10 µg/mL herring sperm DNA for TaqMan assays. Absolute quantifications of 16S rDNA, *sul1*, *sul2*, *qnrA*, *qnrB*, *qnrS*, and *Enterococcus* spp. 23S rRNA genes were performed with serial diluted exogenous standards that consisted of purified PCR products. The standards were generated by specific PCR with reference strains which contain the target gene. We used the following reference strains: *Enterococcus faecium* E155 (clinical isolate) [1] for *Enterococcus* spp. 23S rRNA gene and bacterial 16S rRNA gene, *E. coli* Hm06-20 encoding *qnrA1* and *sul1*, *E. coli* PS84 encoding *qnrS1* and *sul2*, and *Klebsiella pneumoniae* K8-5 encoding *qnrB1*. Primers and probes applied in qPCR (and PCR for generating standards) are listed in Table S4 of the SI. Oligonucleotides were purchased from Sigma-Aldrich (Sigma-Aldrich, Steinheim, Germany) and TaqMan probes were purchased from TIB Molbiol (Berlin, Germany). Each 50 µL PCR reaction consisted of 5 µL reaction buffer (Peqlab, Erlangen, Germany), 2 mM MgCl_2_, 0.2 µM of each primer, 2.5 U Taq polymerase, 0.2 mM of each dNTP (Peqlab, Erlangen, Germany), and about 100 ng of template DNA. PCR was carried out with the following three segments: (i) initial denaturation at 95°C for 2 min, (ii) 30 cycles of 95°C for 30 s, 30 s at temperatures specified in Table S5, 72°C for 2 min, and (iii) elongation at 72°C for 10 min. PCR products were purified with QIAquick Gel Extraction Kit (Qiagen, Hilden, Germany).

Quantification of absolute target gene numbers was carried out using the Light-Cycler 480 (Roche Diagnostics, Mannheim, Germany). TaqMan probes were labeled at the 5´ end with FAM and at the 3´ end with TAMRA. We used serial dilutions (10, 100, 1000, 10,000, 100,000, 1000,000 10,000000, 50,000000 gene copies/reaction) of gel-purified PCR products as templates for generation of standard curves for resistance and bacterial genes. DNA was diluted with Tris-EDTA buffer (10 mM Tris, 1 mM EDTA, pH 8) for SYBR Green I qPCR or Tris-EDTA buffer (10 mM Tris, 1 mM EDTA, pH 8) containing 10 µg/mL herring sperm DNA for TaqMan assays. The limit of quantification (LOQ) for *sul1*, *sul2*, *qnrB*, and *qnrS* was 10 gene copies/reaction. For 16S rDNA, *Enterococcus* spp. and *qnrA* the limit of quantification equaled 100 gene copies/reaction. Reagents and programs for qPCR are listed in Table S5.

**REFERENCES**

1. Kropec A, Sava IG, Vonend C, Sakinc T, Grohmann E, et al. (2011) Identification of SagA as a novel vaccine target for the prevention of *Enterococcus faecium* infections. Microbiology-Sgm 157: 3429-3434.
